# A large net carbon loss attributed to anthropogenic and natural disturbances in the Amazon Arc of Deforestation

**DOI:** 10.1073/pnas.2310157121

**Published:** 2024-08-05

**Authors:** Ovidiu Csillik, Michael Keller, Marcos Longo, Antonio Ferraz, Ekena Rangel Pinagé, Eric Bastos Görgens, Jean P. Ometto, Vinicius Silgueiro, David Brown, Paul Duffy, K. C. Cushman, Sassan Saatchi

**Affiliations:** ^a^Jet Propulsion Laboratory, California Institute of Technology, Pasadena, CA 91109; ^b^International Institute of Tropical Forestry, United Stated Department of Agriculture (USDA) Forest Service, Río Piedras 00926, Puerto Rico; ^c^Climate and Ecosystem Sciences Division, Lawrence Berkeley National Laboratory, Berkeley, CA 94720; ^d^College of Forestry, Oregon State University, Corvallis, OR 97333; ^e^Department of Forest Engineering, Universidade Federal dos Vales do Jequitinhonha e Mucuri, Diamantina, MG 39100-000, Brazil; ^f^Earth System Sciences Center, National Institute for Space Research-National Institute for Space Research (INPE), São José dos Campos, SP 12227-010, Brazil; ^g^Instituto Centro de Vida, Alta Floresta, MT 78580-000, Brazil; ^h^Neptune and Company, Inc., Lakewood, CO 80215; ^i^Environmental Sciences Division, Oak Ridge National Laboratory, Oak Ridge, TN 37830

**Keywords:** Amazon, tropical forest, carbon loss, forest disturbance, airborne lidar

## Abstract

The Amazon forest contains globally important carbon stocks, but in recent years, atmospheric measurements suggest that it has been releasing more carbon than it has absorbed because of deforestation and forest degradation. Accurately attributing the sources of carbon loss to forest degradation and natural disturbances remains a challenge because of the difficulty of classifying disturbances and simultaneously estimating carbon changes. We used a unique, randomized, repeated, very high-resolution airborne laser scanning survey to provide a direct, detailed, and high-resolution partitioning of aboveground carbon gains and losses in the Brazilian Arc of Deforestation. Our analysis revealed that disturbances directly attributed to human activity impacted 4.2% of the survey area while windthrows and other disturbances affected 2.7% and 14.7%, respectively. Extrapolating the lidar-based statistics to the study area (544,300 km^2^), we found that 24.1, 24.2, and 14.5 Tg C y^−1^ were lost through clearing, fires, and logging, respectively. The losses due to large windthrows (21.5 Tg C y^−1^) and other disturbances (50.3 Tg C y^−1^) were partially counterbalanced by forest growth (44.1 Tg C y^−1^). Our high-resolution estimates demonstrated a greater loss of carbon through forest degradation than through deforestation and a net loss of carbon of 90.5 ± 16.6 Tg C y^−1^ for the study region attributable to both anthropogenic and natural processes. This study highlights the role of forest degradation in the carbon balance for this critical region in the Earth system.

## Significance

This study presents a detailed partitioning of aboveground carbon losses and gains in the Amazon forest, illuminating the critical role of forest degradation in the regional carbon balance. Using high-resolution airborne laser scanning, we quantified the impacts of human activities and natural disturbances on carbon loss. Forest degradation through logging and fires directly impacted 3.5% of the surveyed area surpassing the area of forest cleared (0.7%). Our findings indicate that the Brazilian Arc of Deforestation experienced a net annual carbon loss of −90.5 ± 16.6 Tg C y^−1^ between 2016 and 2018 further highlighting the importance of forest degradation for the carbon budget of this critical region in the Earth system.

Tropical forests are vital to combating climate change because they absorb and store more aboveground carbon than any other terrestrial ecosystem (1). However, human activities and recent changes in regional climate have caused significant changes to the structure, integrity, and biodiversity of these forests (2–5). In particular, the Brazilian Amazon has experienced severe deforestation and degradation, leading to the region becoming a carbon source rather than a sink (6–8). While the effects of deforestation on carbon loss have been thoroughly researched (7, 9, 10), the carbon impact of forest degradation is not well understood and is difficult to quantify accurately at a large scale (11–14). Degradation is more spatially dispersed than deforestation, expanding the frontiers of forest loss (15). Degradation is often a precursor of deforestation, with almost half of the degraded tropical forests being cleared in subsequent years (15, 16). Carbon emissions from forest disturbances in the Amazon are equivalent to, if not greater than, the emissions from deforestation although the range of current estimates is very wide (0.05 to 0.2 Pg C y^−1^) (12). The vulnerability of tropical forests to climate change, including more frequent and severe droughts, as well as increased susceptibility to fires, further intensifies the degradation of these forests, resulting in accelerated carbon losses and ecosystem disruptions (4, 17, 18).

Anthropogenic forest degradation, caused by selective logging, forest fires, and fragmentation, reduces tree cover without completely removing it (19). Selective logging harvests merchantable tree species using a network of roads that provide access for machinery (20, 21). Harvesting practices often result in high levels of canopy damage, contributing to forest fire vulnerability (22, 23). Forest fires in the Amazon forest are nearly all ignited by humans (24). Forest fires affected 16.4% of the Amazon biome between 1985 and 2020 (25) and are projected to intensify due to changing climate (26). Severe droughts increase fire occurrence leading to increased fire-induced tree mortality observed recently (27–29). Forests degraded by logging and fires may contain less than half of the carbon stocks in intact forests (13, 30, 31) while associated forest edges and fragmentation effects promote indirect carbon losses (32, 33). The recovery of degraded carbon stocks to levels similar to intact forest may take decades (30, 34) but can partly counterbalance carbon emissions from forest loss (35, 36).

Natural forest disturbances are dominated by small-scale mortality events (<0.1 ha) (37). The Amazon biome is experiencing increasing mortality of individual trees within intact and old-growth forests (38). Larger natural disturbances such as windthrows create gaps of uprooted or broken trees (39). A strong correlation exists between the occurrence of windthrows and frequency of heavy rainfall (40), with windthrows concentrated mostly in central and northwestern Amazon (41, 42). The windthrow disturbances are predicted to increase under warming climate scenarios (43).

Despite recent efforts to quantify the carbon losses and gains from forest degradation and recovery, the estimates remain highly variable (12, 13, 44). Field inventory data, which are often limited to intact forests (45, 46) and rarely designed to cover areas with human disturbance (34, 47), provide a limited sample of plots due to accessibility and cost (48). Satellite-based approaches, despite their wider coverage, suffer from coarse resolution that makes it difficult to quantify the extent and intensity of forest degradation because the signal of selective logging and fires fades away between cloud-free observations due to regeneration (49, 50). Furthermore, forest degradation is heterogeneous not only in space and time but also in intensity (30, 51), which makes its unambiguous detection challenging. Repeated airborne lidar (light detecting and ranging) can accurately detect changes in forest structure between different acquisitions and has been used to estimate carbon dynamics due to forest degradation, however, its application has been limited to isolated case studies (52, 53).

Here, we estimate changes of forest aboveground carbon (AGC) stocks attributed to both human-induced degradation and natural disturbances, and the postdisturbance regrowth over an area of active land use change in the southern Brazilian Amazon. We directly measure changes in canopy heights using a randomized sample of 99 repeated airborne lidar scanning (ALS) transects (~500 ha each) covering forests in the Brazilian Arc of Deforestation between 2016 and 2017 to 2018. We provide a unique and detailed quantification of canopy structural change that accounts for both land cover processes and aboveground carbon density (ACD) changes. Our approach permits direct estimation of rates of AGC changes due to forest clearing, selective logging, fires, windthrows, and other disturbances, as well as forest growth. We apply our findings to the Arc of Deforestation and explore the importance of territorial protection for changes in carbon storage.

## Results

**Fig. 1.**
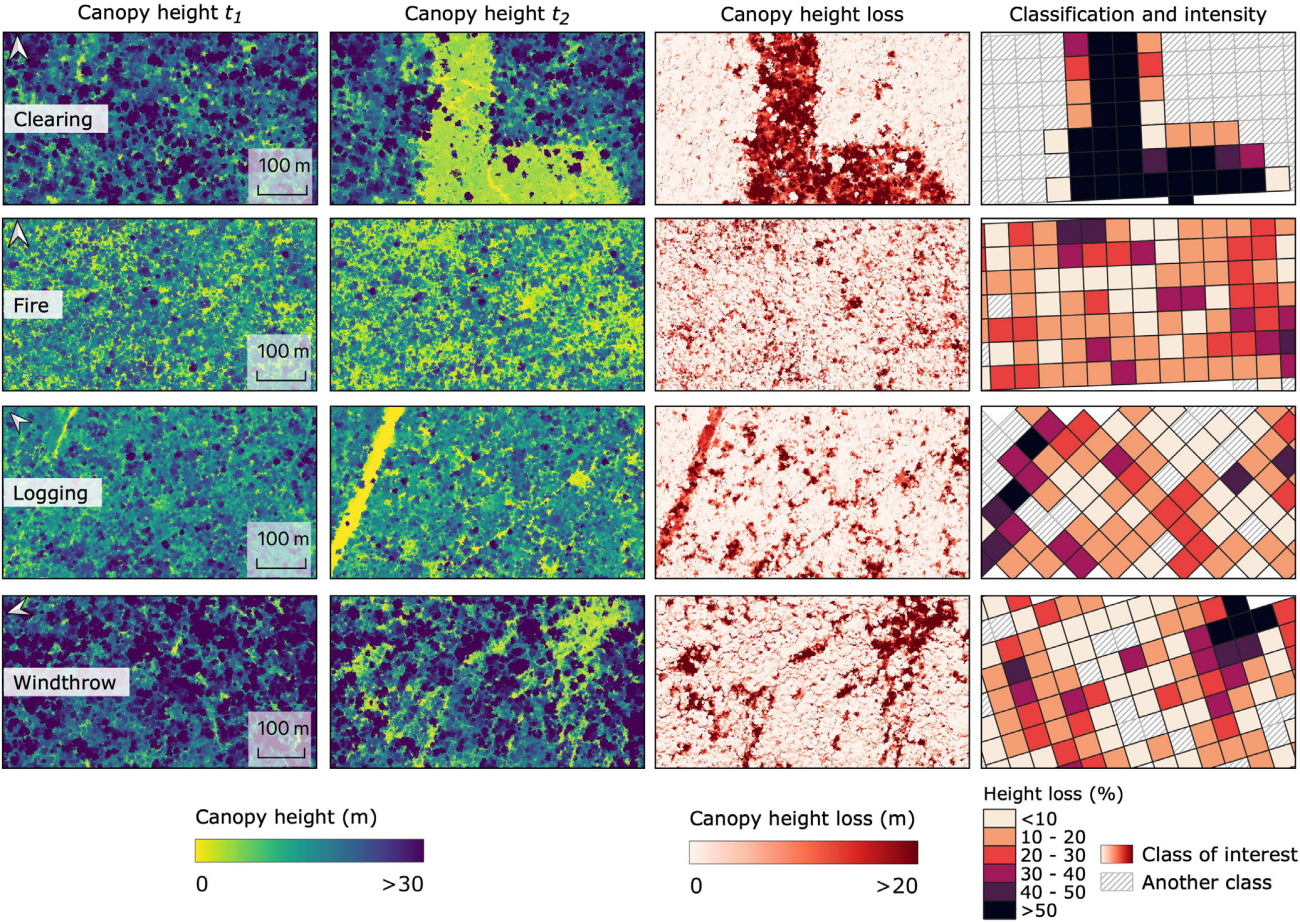
Canopy height loss calculated from lidar time-series and the classification (clearing, fire, logging, and windthrow) based on 50 × 50 m cells. The first two columns show the canopy height from the first (*t_1_*) and second (*t_2_*) airborne lidar campaigns. The third column displays the canopy height loss in the period between the two acquisitions. The fourth column shows the cells identified as one of the four disturbance events and the intensity of relative height loss.

**Fig. 2.**
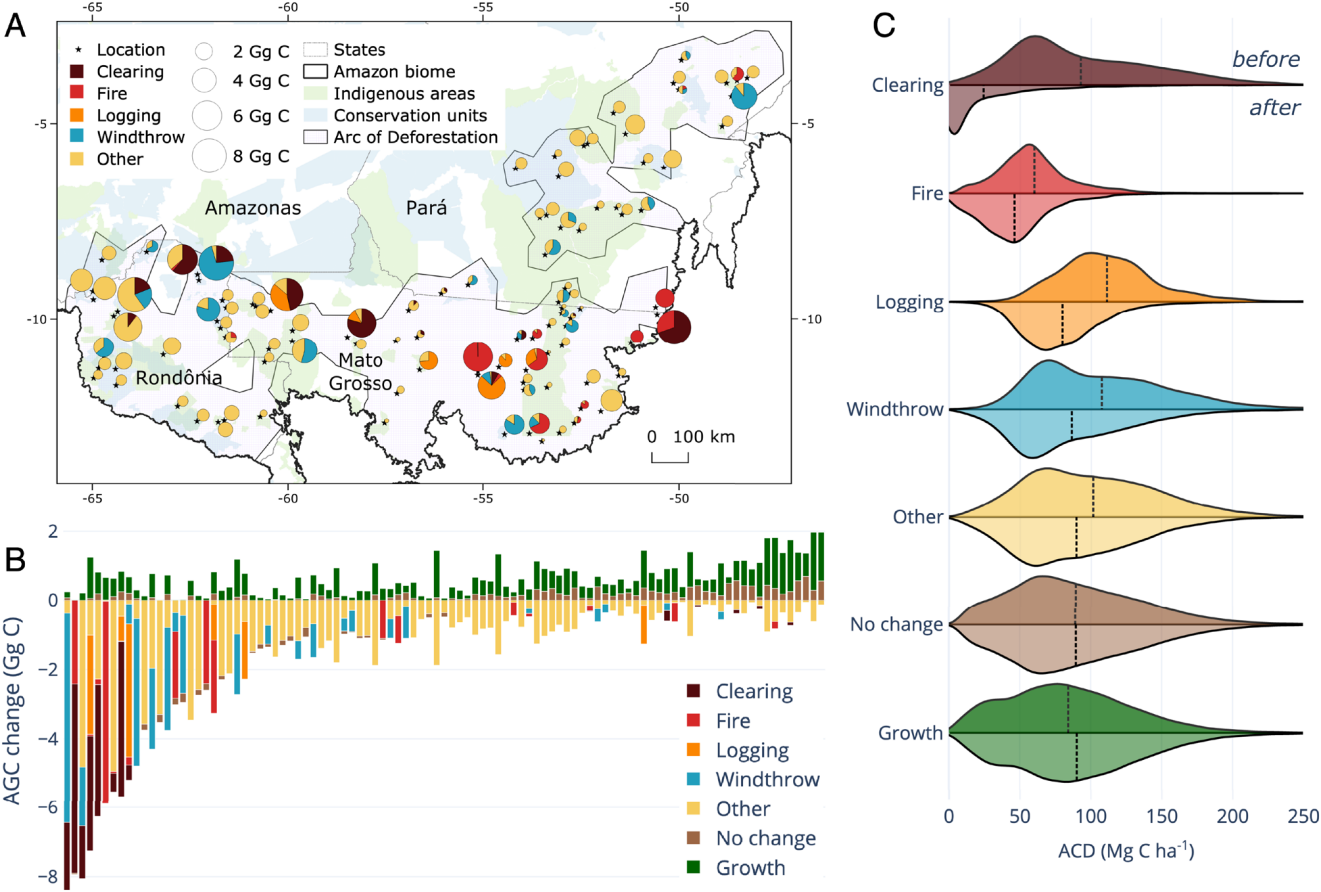
Spatial distribution and transect-wise statistics of AGC dynamics. (*A*) Random locations of repeated lidar transects in the Brazilian Amazon biome sampled in 2016 and 2017 to 2018. Each pie chart shows the carbon loss contribution of the five forest disturbance classes within each transect, with the size of the circle proportional to the gross AGC loss due to clearing, fire, logging, windthrow, and other disturbances (range 0.005 to 8.4 Gg C). (*B*) Bar chart showing the AGC change (Gg C) for each of the seven classes analyzed and for each of the 99 transects, ordered by the net AGC change. (*C*) Variation in the overall distribution of aboveground carbon density (ACD, Mg C ha^−1^) between the two lidar campaigns. The first campaign is shown on top and the second campaign is shown below the line for each class. The black dotted vertical lines represent the mean ACD.

We analyzed 48,280.25 ha of forest in 99 transects using repeated airborne lidar and found that 4.2% of the area registered forest height loss clearly attributable to human activity in the period between the lidar campaigns, including clearing (0.7%), logging (0.7%), and forest fires (2.8%). Windthrows disturbed 2.7% of the surveyed forest, while other small natural and anthropogenic disturbances affected 14.7% (*SI Appendix*, Fig. S1). The canopy loss classes follow distinct spatial patterns as shown in the lidar canopy height time-series (Fig. 1). The distribution of forest disturbances across the Arc of Deforestation was heterogeneous (Fig. 2*A*). Selective logging and fires predominantly occurred in the state of Mato Grosso, while clearing pushed forward the deforestation frontiers in Rondônia and Mato Grosso. Windthrows and other canopy disturbances were distributed throughout the entire Arc of Deforestation. Detectable forest growth (≥0.5 m) covered 16.3% of the area, while 62.1% of the forest did not change more than our conservative detection threshold (absolute change < 0.5 m) (*SI Appendix*, Fig. S1).

We estimated ACD for 193,121 cells of 0.25 ha using a parametric model based on the lidar mean top of canopy height (TCH) (31). For the 99 transects, 64 experienced a net AGC loss, while 35 had a net AGC gain (Fig. 2*B*). Only 10 transects accounted for 57.4% of the net AGC loss with a mixture of anthropogenic and natural disturbances (Fig. 2*B*).

We observed different patterns of variation in the overall distribution of ACD for the seven canopy change classes analyzed (Fig. 2*C*). Forest fires affected areas with the lowest mean ACD in 2016 (60.0 Mg C ha^−1^). The 17 transects with fire events were more fragmented and had less forest area (80.7%) than the other 82 transects not affected by fire (91.1% forest area), had higher accessibility through roads (mean proximity to roads is 34.2 km, compared with 147.1 km), and suffered greater seasonally water stress (climatic water deficit of 400.1 mm/y, compared to 349.6 mm/y). Forests that were selectively logged had the highest mean ACD in 2016 (111.6 Mg C ha^−1^), while clearing led to the greatest mean ACD reduction (Fig. 2*C*). Clearing led to a mean ACD loss of −68.3 ± 13.3 Mg C ha^−1^ (a decrease of 73.8% from initial ACD), logging −31.8 ± 5.2 (28.5% loss), windthrow −21.7 ± 5.1 (20.1% loss), fire −13.9 ± 2.9 (23.2% loss), and other −11.9 ± 0.8 (11.7%), while no change had ACD gain of 0.4 ± 0.2 (0.5% gain) and growth of 6.2 ± 0.4 Mg C ha^−1^ (7.3% gain).

**Fig. 3.**
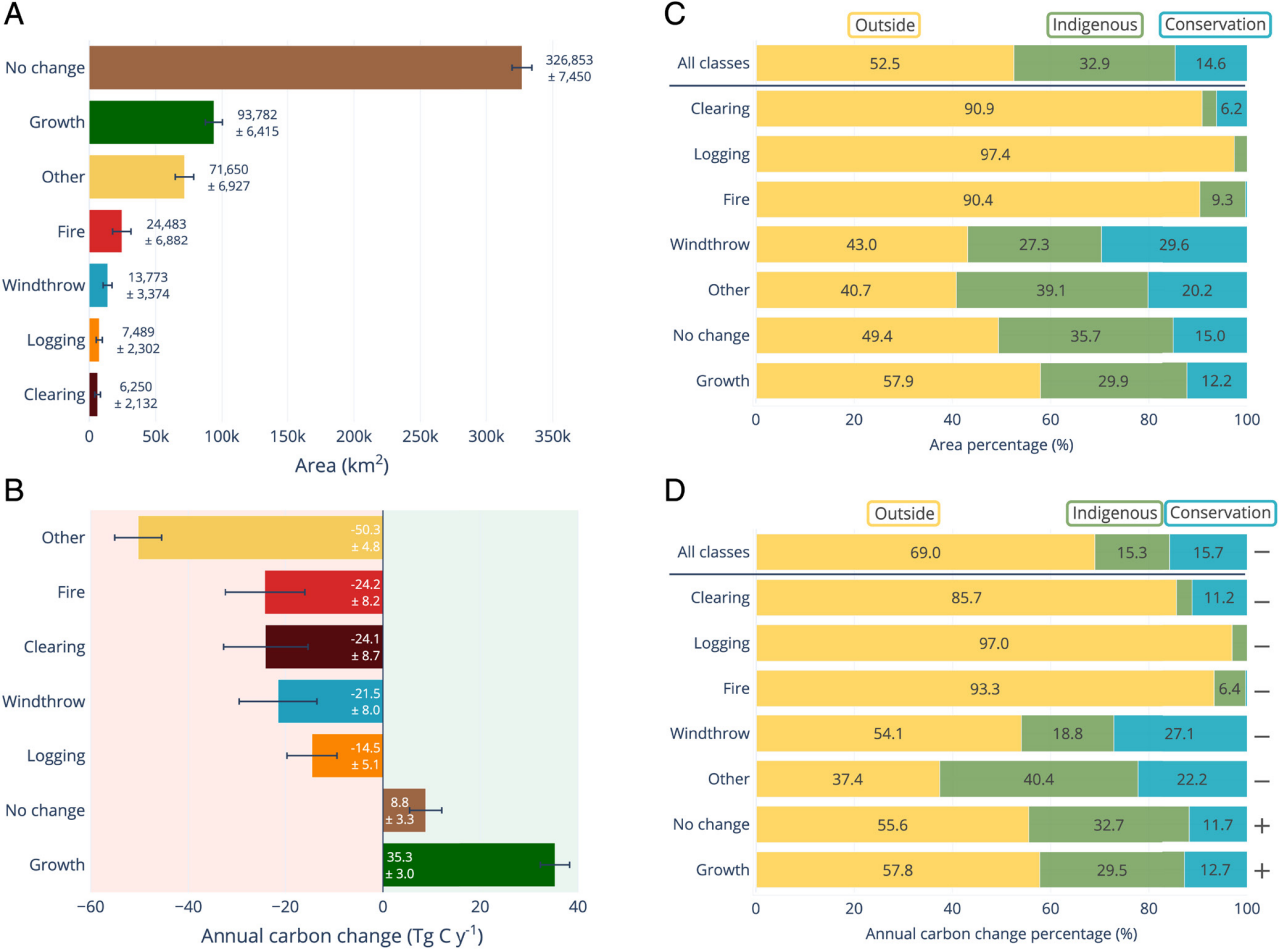
Extrapolation statistics for the Brazilian Arc of Deforestation (544,300 km^2^). The extrapolation was stratified based on protection status: indigenous territories, conservation units, and outside these two areas. (*A*) Total area (km^2^) and (*B*) Annual aboveground carbon change (Tg C y^−1^) for each of the seven classes between the two lidar campaigns. Error bars represent the SD obtained by combining prediction, carbon estimations, and classification uncertainties, respectively (*Materials and Methods*). (*C*) Area percentage (%) and (*D*) annual aboveground carbon change percentage (%) for each class within each of the three protection statuses. The minus and plus signs in (*D*) represent carbon loss and gain, respectively.

We scaled our lidar-based area and carbon statistics to the forest area in the Arc of Deforestation (544,300 km^2^) (Fig. 2*A*), stratifying the extrapolation based on the areas and carbon dynamics within indigenous territories, conservation units, and outside these protected areas that we estimated from our lidar statistics for each of the seven classes. Selective logging affected 7,489 ± 2,302 km^2^, fires 24,483 ± 6,882 km^2^, and clearing 6,250 ± 2,132 km^2^. The total area attributed to forest growth was comparable to that of windthrows and other disturbances, combined (Fig. 3*A*). Surprisingly, we found that windthrows had a comparable annual AGC gross loss (−21.5 ± 8.0 Tg C y^−1^) to clearing (−24.1 ± 8.7 Tg C y^−1^) and fire (−24.2 ± 8.2 Tg C y^−1^) and was 148% of selective logging (−14.5 ± 5.1 Tg C y^−1^). The other types of forest disturbance accounted for the highest annual AGC change (−50.3 ± 4.8 Tg C y^−1^), while +35.3 ± 3.0 Tg C y^−1^ was sequestered through forest growth and recovery and +8.8 ± 3.3 Tg C y^−1^ through net small growth in the area that we classified as no change (Fig. 3*B*). Summing the annual AGC changes of all extrapolated categories resulted in an annual net AGC change of −90.5 ± 16.6 Tg C y^−1^ or −99.3 ± 16.3 Tg C y^−1^ when the no change category is excluded (Fig. 3*B*).

We found that indigenous territories and conservation units were effective in protecting the forest against anthropogenic degradation. While combined, they occupied 47.5% of the area in our-defined Arc of Deforestation, they only contained 9.1% of clearing, 2.6% of logging, and 9.6% of fires (Fig. 3*C*). Clearing, logging, and fires outside these two protected lands had an annual AGC loss of −57.3 ± 12.9 Tg C y^−1^, while conservation units had −2.8 ± 1.5 Tg C y^−1^ and indigenous territories had −2.7 ± 1.0 Tg C y^−1^ for the same three classes (Fig. 3*D*).

## Discussion

Using the most extensive, repeated high-resolution airborne lidar data in the Amazon Arc of Deforestation (544,300 km^2^) to date, we found an annual net AGC loss of −90.5 ± 16.6 Tg C y^−1^ between 2016 and 2017 to 2018. Forest degradation and natural disturbances affected an area of 79,721 km^2^ y^−1^, greater than a previous estimate of 60,000 km^2^ between 2016 and 2017 for the entire Brazilian Amazon (54). Specifically, forest degradation and disturbances accounted for 82.1% of the annual gross AGC losses, surpassing the findings of Qin et al. (55) and Fawcett et al. (35), who reported percentages of 73% and 79%, respectively. We found that indigenous territories and conservation units protected the forest against anthropogenic carbon losses (clearing, logging, fire), in agreement with previous studies (56, 57). Even so, these protected areas are under threat from illegal loggers, miners, ranchers, farmers, infrastructure projects, and escaping fires (26, 58), with increasing deforestation rates during 2013 to 2021 (59).

We estimated 4,616 ± 2,302 km^2^ y^−1^ of selective logging for the Arc of Deforestation, comparable with the estimate of 5,738 km^2^ y^−1^ reported for the Brazilian Amazon between 2010 and 2014 (15) and the estimate of 7,041 km^2^ y^−1^ for the entire Amazon (6.6 M km^2^) between 2001 and 2018 (12). Furthermore, our findings indicate that logged forests lost −31.8 ± 5.2 Mg C ha^−1^ between 2016 and 2017 to 2018 (mean 111.6 Mg C ha^−1^ in 2016). This loss is greater than the losses of 9.0 Mg C ha^−1^ (52) and 10.4 Mg C ha^−1^ (53) estimated for experimental sites of reduced-impact logging with minimal collateral damages, as assessed using repeated airborne lidar measurements. However, our estimated loss was lower than the ACD loss of 37.1 Mg C ha^−1^ (60) and 51.2 Mg C ha^−1^ (30) for logged forests compared to undisturbed forest in regions highly degraded by logging activities, calculated using forest inventory plots and a chronosequence of lidar samples, respectively.

We identified 16,419 ± 6,882 km^2^ y^−1^ of forests that burned in our defined Arc of Deforestation. This value is two times higher than the estimate of 8,237 km^2^ y^−1^ for the entire Brazilian Amazon biome (4.2 M km^2^) between 1985 and 2020 (25) and ten times higher than the estimate of 1,522 km^2^ y^−1^ for the burned forest area in the Brazilian Amazon between 1992 and 2014 (15). Moreover, our findings indicate that our estimated burned forest area is two times higher than 7,213.2 km^2^ y^−1^ estimated for the entire Amazon (6.6 M km^2^) between 2001 and 2018 (12). Forest fires resulted in a reduction of ACD with 23.2%, equivalent to −13.9 ± 2.9 Mg C ha^−1^. This loss is lower than previously reported values of 67.7 Mg C ha^−1^ (30) and 51.5 Mg C ha^−1^ (18). We estimated carbon losses of 24.2 ± 8.2 Tg C y^−1^ from forest fires, which is higher than 10.3 ± 11.3 Tg C y^−1^ estimated for the Brazilian Amazon between 2003 and 2015 (27). We attribute the greater estimation of burned areas to the use of very high-resolution airborne lidar data in our study, which is more sensitive to detecting damage from understory fires compared to satellite-based detection of fires. Furthermore, the lidar sampling was conducted between 2016 and 2018, a period reported to have higher than average disturbance rates following the 2015 El Niño drought (16, 35). Morton et al. (61) also found temporal concentration of understory forest fires (>50 ha) in three years between 1999 and 2010 affecting 2.8% of all forest, mainly when severe droughts occurred.

Windthrows affected 9,638 ± 3,374 km^2^ y^−1^ in our study, resulting in AGC losses of 21.5 ± 8.0 Tg C y^−1^ and indicating that windthrows may be more common and widespread in the Amazon than previously thought. Earlier estimates suggested that windthrows accounted for a carbon loss of only 3 Tg C y^−1^ for the entire Amazon forest area (6.8 M km^2^) (37). However, our detailed study identified 24 windthrow events with a minimum size of 0.35 km^2^ in a randomly sampled forest area of 482.8 km^2^ across Southern Amazonia. Although previous studies suggest that our sampled region has a low density of windthrow events (43), we found a higher density compared to the state of Amazonas, where density was estimated to reach 12 events per 10,000 km^2^ between 2018 and 2019 for events larger than 0.025 km^2^ (43). Similar to fires, windthrows may have an interannual variability and we might have sampled a year with strong convective storms following the extreme drought in 2015, and further studies are needed to better understand the weather and climate conditions that favor windthrow events, as well as their ecological impact and significance.

We found that forest accumulated AGC at a rate of 3.8 Mg C ha^−1^ y^−1^ for all grid cells where height increase exceeded 0.5 m between the two lidar campaigns (*SI Appendix*, Fig. S2). The no change class, characterized by small canopy height losses and gains smaller than 0.5 m, exhibited a net AGC accumulation rate of 0.3 Mg C ha^−1^ y^−1^. This suggests that minor growth outweighed minor losses in the no change class (*SI Appendix*, Fig. S3). Both the growth and the no change categories may include areas recovering from prior degradation or secondary forests where we would expect faster rates of biomass accumulation than in intact forest (34, 62). Carbon accumulation in our growth category is consistent with previous estimates for second growth for South American moist forests, with carbon accumulation rates ranging from 1.3 to 5.5 Mg C ha^−1^ y^−1^ as reported in several studies (13, 34, 36, 62–68).

Aspects of the current study may lead to under- or overestimation of AGC changes. Our lidar sampling was limited to forested areas, which may have undersampled edges and smaller fragmented forests. As edges and forest fragments contribute significantly to carbon losses (15, 32), we may have underestimated annual net AGC loss. We classified as other disturbances events that could not be identified with high certainty, such as natural branch and tree mortality including drought mortality; edge effects; postlogging and postfire mortality; isolated blowdowns, diffuse windthrow effects, flooding, landslides, or clandestine logging (*SI Appendix*, Fig. S4). We mapped the forest disturbance events and growth that happened between the first and second airborne campaigns. Expanding the analysis to investigate the history and legacy of the forest disturbances over longer time periods will help to better understand the long-term impact on carbon storage and recovery capacity of disturbed forests.

We quantified multiple sources of uncertainty with assumptions that can potentially lead to over- or underestimation of the uncertainty of AGC estimates (*Materials and Methods*). Overall, we aimed to make our uncertainty estimates of AGC conservative and to integrate major sources of uncertainty given the complexity of our data and the stratified estimation approach used to extrapolate lidar-based estimates to the Brazilian Arc of Deforestation.

Forest degradation is often difficult to quantify and monitor, because it occurs in subtle ways that are not easily detectable through conventional remote sensing methods and in places where access on the ground may be controlled by landowners conducting illegal or irregular activities. We used the most extensive repeated high-resolution airborne lidar data for the Amazon to capture fine-scale changes in forest structure to demonstrate the importance of aboveground carbon losses due to forest degradation for the regional carbon budget. Our results significantly advance our understanding of degradation in tropical forests and its implications for the carbon budget highlighting the need for improved and continued high-resolution monitoring of forest structure and assessment of forest disturbances and recovery.

## Materials and Methods

### Airborne Lidar Acquisition and Processing.

We analyzed 99 airborne lidar transects that were revisited in two campaigns within the EBA (Estimating the Biomass of the Amazon) project (69). The first campaign acquired data between March and December 2016 and the second campaign took place between November 2017 and April 2018 (*SI Appendix*, Fig. S5). The average time difference between the two campaigns is 596 ± 101 d (mean ± SD), with a minimum difference of 350 d and a maximum of 738 d. The lidar transects were collected using multiple returns from a Trimble Harrier 68i airborne sensor. The pulse footprint area was set to be below 30 cm and the flying altitude was 600 m above ground. To ensure a detailed characterization of terrain elevation, the required average point density for collection was four returns per square meter. Horizontal and vertical accuracy were controlled to be under 1 m and 0.5 m, respectively (69). The 99 transects in 2016 were selected based on a random sampling design, constrained to avoid deforested areas using a deforestation mask derived from PRODES (70) and TERRACLASS (71) products. The transects were positioned by randomly generating center points with X, Y coordinates and azimuth (72). Start points were visually examined to verify that they fell within the forest or secondary vegetation mask. Any start points not within a forest, as identified by satellite imagery, were discarded and replaced. A shapefile containing a polygon measuring 12.5 km × 300 m was created for each point. In case of conflicts with the flight plan (such as proximity to an airport or military restrictions), the flight company requested repositioning to the nearest permitted area. More details about the sampling of airborne lidar transects can be found in Ometto et al. (72).

Several steps were undertaken to process the lidar point clouds and obtain canopy height models (CHMs). We classified ground and off-ground points. We calculated digital terrain models (DTM) and digital surface models (DSM) at 1 m spatial resolution. We normalized the point cloud to obtain vegetation height above ground and calculated CHMs at 1 m spatial resolution by selecting the highest point in each 1-m^2^ cell. For consistency, we calculated the CHMs for each of the two lidar campaigns using the DTM derived from the first lidar campaign. For subsequent analyses, we created a grid of 50 × 50 m (0.25 ha) and kept the cells that had an overlap with the CHM greater than 99% for both campaigns (49,654 ha). We further excluded 1,373.75 ha that were overlapping nonforest in both lidar campaigns, resulting in a total area of 48,280.25 ha across the 99 transects, for which we estimated aboveground carbon changes (Fig. 2). We defined a forest as a minimum area of 1 ha having a minimum canopy cover of 30% and a minimum tree height of 5 m (48). We defined degraded forests as areas where anthropogenic disturbances (fire and logging) resulted in average canopy height loss greater than 0.5 m.

### Classification of Forest Disturbances and Growth.

We classified all 193,121 grid cells of 0.25 ha into one of the seven classes: clearing, selective logging, fires, windthrows, other disturbances, growth, and no change. We calculated the average height change in the CHMs for each 0.25 ha cell from 2,500 height differences at 1 m resolution. We used a conservative threshold average change of 0.5 m (targeted vertical accuracy of the lidar data) when considering whether a cell experienced canopy height loss or gain. Cells with an absolute average height change less than 0.5 m were included in a no change class to avoid measurement artifacts. We delineated forest clearing, selective logging, forest fire, and windthrow events using visual interpretation of time series of Sentinel-2 images and PlanetScope NICFI mosaics (73) in Google Earth Engine (74). These four classes were delineated within all 99 transects, for the period between the first and second airborne lidar campaign. We overlapped these delineations with the 50 × 50 m grid cells and classified the cells into one of these four classes if the canopy height loss was greater than 0.5 m. However, not all disturbance events can be identified through visual interpretation of satellite images. Thus, the other class contains the remaining canopy height losses greater than 0.5 m that did not have a clear attribution of the disturbance driver as seen from the satellite images. This included, but was not limited to, clandestine logging, drought mortality, edge effects, delayed mortality from pre-2016 degradation events, isolated small blowdowns, diffused windthrow effects, flooding, or landslides. Cells with canopy height gains higher than 0.5 m were classified as growth (*SI Appendix*, Fig. S6).

We defined forest clearing as areas where the canopy cover was removed (Fig. 1). We mapped selective logging for areas having clear patterns recognizable as industrial selective logging that rely on networks of roads and trails to provide access for machinery and the transport of merchantable timber products (20). Small-scale logging operations are harder to identify on optical satellite imagery when they use portable mills and plank skidding with animal traction (75). Because we required clear evidence of log storage and transport to identify logging areas, we have conservatively classified logging areas. Forest fires are well distinguished on the near-infrared and short-wave infrared bands of Sentinel-2 images. Forest fires often affect already degraded areas and can have multiple patterns depending on the triggering factor and land management (76). Windthrows were predominantly identified as fan-shaped or diffuse geometry disturbances caused by convective storms (41, 43). We found windthrow events inside intact forests as well as bordering degraded areas, with our transects overlapping different intensity parts of a windthrow.

### Aboveground Carbon Estimates and Changes.

We estimated ACD at 0.25 ha spatial resolution using a parametric model based on the mean top of canopy height (TCH), which makes the model easily transferable because it is insensitive to differences among lidar characteristics (31, 77). The allometric equation was developed using a total of 18 sites covering 18,006 ha in intact, degraded, and regenerating forest types in the Brazilian Amazon, surveyed with forest inventories and multiple-return small-footprint airborne lidar (31):

$${\mathrm{ACD}}_{\mathrm{TCH}}=0.54\times {\mathrm{TCH}}^{1.76},$$

where ACD (Mg C ha^−1^) is aboveground carbon density and TCH (m) is the mean top of canopy height, calculated as the mean of all 1 m pixels of maximum lidar return height within the 50 × 50 m cell (0.25 ha). Model uncertainty estimation is described in detail in a following section.

Annual change in AGC stocks for each 0.25 ha cell, including all disturbance types and growth, was calculated using the stock-difference method (78) and annualized to account for different time intervals between the airborne lidar revisits for each transect:

$$\Delta AGC_{\mathrm{cell}}=\frac{\left({\mathrm{AGC}}_{t2}-{\mathrm{AGC}}_{t1}\right)}{\Delta t}\times 365,$$

where $\Delta AGC_{\mathrm{cell}}$ is the annual change in AGC stocks (Mg C y^−1^) for a 0.25 ha cell, ${\mathrm{AGC}}_{t2}$ and ${\mathrm{AGC}}_{t1}$ (Mg C) are the total AGC stocks at time t_2_ and t_1_, respectively, Δt is the time difference in days (t_2_ − t_1_) between the two acquisition dates of the airborne lidar for that cell.

Annual net AGC change for all lidar transects was calculated using the gain–loss method (78), where gains include AGC sequestration due to forest growth and losses representing the AGC emissions (both instantaneous emissions and committed emissions to the atmosphere following the decomposition of dead organic matter) due to forest degradation and disturbances.

$$\begin{array}{c} \Delta {\mathrm{AGC}}_{\mathrm{annual}}=\Delta C_{\mathrm{Clearing}}+\Delta C_{\mathrm{Fire}}+\Delta C_{\mathrm{Logging}}+\Delta C_{\mathrm{Windthrow}}\\ \;+\Delta C_{\mathrm{Other}}+\Delta C_{\mathrm{No change}}+\Delta C_{\mathrm{Growth}}, \end{array}$$

where $\Delta {\mathrm{AGC}}_{\mathrm{annual}}$ is the annual net AGC change and $\Delta C_{\mathrm{class}}$ is the annual net AGC for each of the seven classes used in this study (Mg C y^−1^). We do not consider changes to coarse or fine litter or belowground carbon in our analyses.

### Extrapolation to the Brazilian Amazon Arc of Deforestation.

The first airborne lidar campaign covered the Brazilian Amazon with >550 randomly selected transects. We used 99 transects that were repeated in a second airborne campaign, concentrated in the states of Rondônia (23 transects), Mato Grosso (42 transects), Pará (32 transects), and southern Amazonas (2 transects). We generated Voronoi polygons for all transects to delineate the domain represented by our repeated sampled lidar transects. We refer to this domain as the Arc of Deforestation, a term we use to refer to the forested area represented by our sample in the southern Brazilian Amazon. The Arc of Deforestation lacks a consistent definition. Voronoi polygons were solely used for delineating the Arc of Deforestation and were not used or considered in any other part of the analysis. This resulted in a total domain area of 855,500 km^2^, from which forest covered 544,300 km^2^ (63.6%), according to MapBiomas classification in 2016 (79).

The distribution of our transect area was 54.2% in indigenous territories, 19.4% in conservation units, and 26.4% outside protected areas. A small percentage of transects (1.9%) overlapped shared areas between indigenous territories and conservation units, and this was treated as part of the indigenous territories for analyses. Because the land uses and therefore the annual rates of AGC changes are different depending on the protection status of the location of transects, we performed a stratified extrapolation to our-defined Arc of Deforestation based on land protection status. Forested areas of the Arc of Deforestation to which we extrapolated the statistics overlapped 32.9% with indigenous territories (including 2.8% overlap with conservation units), 14.6% with conservation units, and 52.5% outside these two types of land protection (Fig. 3C). We assume that the carbon dynamics observed in our sampled areas are indicative of those across the entire Arc of Deforestation, given the diverse representation of disturbance and protection classes along randomly selected transects. As in many large-scale forest lidar surveys, the airborne campaigns were flown without any account of the stratification (80, 81). We performed a poststratification based on the classification of forest change and protection status. In the uncertainty analysis below, we refer to this as stratification. We performed a standard stratified estimation approach, where the estimated stratum mean is multiplied by the stratum area to obtain a stratum total. The stratum totals are then added to obtain an overall estimate.

### Uncertainty Analysis.

We estimated uncertainty in predicted ACD and AGC change at five spatial extents: cells, stratification classes (seven disturbance and growth strata per each of three protection strata, for 21 total stratification classes), disturbance and growth (7 strata), land protection (3 strata), and the Arc of Deforestation. Uncertainty estimates follow model-based inference approaches (82–84).

Total uncertainty (${\hat{\sigma }}_{\mathrm{Cell}}$) is the square root of the variance in predicted ACD for 0.25 ha cells and was estimated by considering three contributions to uncertainty and assuming independence across cells:

$${\hat{\sigma }}_{\mathrm{Cell}}^{2}={\hat{\sigma }}_{\mathrm{CellPU}}^{2}+{\hat{\sigma }}_{\mathrm{CellRV}}^{2}+{\hat{\sigma }}_{\mathrm{CellCU}}^{2},$$

where ${\hat{\sigma }}_{\mathrm{CellPU}}$ is prediction uncertainty (uncertainty associated with imperfect available data for ACD model calibration/validation), ${\hat{\sigma }}_{\mathrm{CellRV}}$ is residual variability (uncertainty associated with residual variation between training data and model predictions), and ${\hat{\sigma }}_{\mathrm{CellCU}}$ is calibration uncertainty associated with the forest inventory estimates used for ACD model calibration/validation (31, 83, 84). We did not include the term included in Cushman et al. (84) for cell-level uncertainty from residual spatial autocorrelation because our ACD model was calibrated using field inventory plots of the same size as cell predictions (0.25 ha). Prediction uncertainty was estimated by bootstrap resampling of calibration plots, where ${\hat{\sigma }}_{\mathrm{CellPU}}$ was the uncertainty across bootstrapped predictions, as described in Longo et al. (31) (called representativeness uncertainty in that analysis). Residual variability was estimated using the heteroskedastic model residuals [called prediction uncertainty in (31)]. Calibration uncertainty was estimated considering both measurement error in inventory plots (tree diameter, tree height, and wood density) and allometric model uncertainty and propagating this uncertainty through the model using a normal uncertainty factor (31). Assuming independence between 0.25 ha cells may lead to underestimation for ${\hat{\sigma }}_{\mathrm{CellPU}}$, while including ${\hat{\sigma }}_{\mathrm{CellCU}}$ may lead to uncertainty overestimation.

We estimated uncertainty in ACD change at the 0.25 ha cell level (${\hat{\sigma }}_{Cell\_Change}$) by combining the uncertainty in the initial (${\hat{\sigma }}_{Cell\_Initial}$) and final ACD estimates (${\hat{\sigma }}_{Cell\_Final}$) and assuming independence between the initial and final measurements of each cell, which may lead to overestimation of the total uncertainty:

$${\hat{\sigma }}_{Cell\_Change}^{2}={\hat{\sigma }}_{Cell\_Initial}^{2}+{\hat{\sigma }}_{Cell\_Final}^{2}.$$

Total uncertainty in AGC for stratification classes, ${\hat{\sigma }}_{\mathrm{Class}}$, in Tg C y^−1^, was calculated based on the change estimates by considering three contributions to uncertainty:

$${\hat{\sigma }}_{\mathrm{Class}}^{2}={\hat{\sigma }}_{\mathrm{ClassSU}}^{2}+{\hat{\sigma }}_{\mathrm{ClassCV}}^{2}+{\hat{\sigma }}_{\mathrm{ClassCU}}^{2}\;,$$

where  ${\hat{\sigma }}_{\mathrm{ClassSU}}$ is sampling uncertainty and ${\hat{\sigma }}_{\mathrm{ClassCV}}$ is cell variability (cell-level ACD uncertainty propagated to class-level estimates). At the spatial resolution of canopy change classes, we also conservatively chose to also represent classification uncertainty associated with the classification into seven forest change classes, ${\hat{\sigma }}_{\mathrm{ClassCU}}$ (85). We did not include a term for residual spatial autocorrelation within change classes because this term is usually negligible compared to prediction uncertainty and residual variability at broad scales (83), supported by the lack of spatial correlation in AGC for 50 m cells previously found for tropical forests (86), and lack of significant residual spatial autocorrelation beyond ~30 m in lidar-based ACD models from numerous ecoregions (84). Sampling uncertainty, ${\hat{\sigma }}_{\mathrm{ClassSU}}$, is associated with the limited number of lidar transects and was estimated using a bootstrap, sampling among transects with replacement. Cell variability, ${\hat{\sigma }}_{\mathrm{ClassCV}}$, was also propagated from cell-level estimates using a bootstrap approach, resampling the AGC estimate for each cell by drawing from a random normal distribution with mean equal to predicted AGC and SD equal to ${\hat{\sigma }}_{Cell\_Change}$. Classification uncertainty, ${\hat{\sigma }}_{\mathrm{ClassCU}}$, was estimated using a probabilistic confusion matrix for our classification (*SI Appendix*, Table S1), based on independent validation data (*SI Appendix*, Tables S2 and S3). We performed a third bootstrap, where at each resample and for each class, we assigned cells to each class based on the probabilities pulled from the confusion matrix of our classification. The uncertainty for each of the three components was quantified by calculating the SD among replications of the total AGC for each class. Each of the three bootstrap analyses consisted of 10,000 iterations, with samples drawn with replacement.

Estimates of uncertainty in total annual AGC for different strata, ${\hat{\sigma }}_{\mathrm{Combined}}$ (in Tg C y^−1^), were predicted by combining the uncertainties of *H* different stratification classes within each stratum, where ${\hat{\sigma }}_{\mathrm{ClassH}}$ is calculated as described above.

$${\hat{\sigma }}_{\mathrm{Combined}}^{2}={\hat{\sigma }}_{Class1}^{2}+{\hat{\sigma }}_{Class2}^{2}+\cdots +{\hat{\sigma }}_{\mathrm{ClassH}}^{2},$$

where *H* = 3 for land protection strata, *H* = 7 for forest change classification strata, and *H* = 21 for the Arc of Deforestation. Each ${\hat{\sigma }}_{\mathrm{ClassH}}$ included the three components of uncertainty (sampling uncertainty, cell variability, and classification uncertainty).

Estimates of uncertainty in mean ACD change (in Mg C ha^−1^) for strata (e.g., disturbance and growth classes, protection, Arc of Deforestation), ${\hat{\sigma }}_{\mathrm{Str}}$, were predicted using a stratified estimator approach, which considers different areas of subgroups within strata when propagating total uncertainty (82):

$${\hat{\sigma }}_{\mathrm{Str}}^{2}=\sum_{h=1}^{H}w_{h}^{2}\cdot {\hat{\sigma }}_{{\mathrm{Class}}_{h}}^{2},$$

where *h = 1, 2, …, H* indexes a stratum, $w_{h}$ is the stratum weight calculated as the proportion of the study area in the *h^th^* stratum, and ${\hat{\sigma }}_{{\mathrm{Class}}_{h}}$ is the estimated mean ACD change uncertainty for the *h^th^* stratum, in Mg C ha^−1^ (82). For this calculation, we assume that the stratum weights, $w_{h}$, are known with certainty. The stratum’s classification uncertainty is already included as a component of the ${\hat{\sigma }}_{{\mathrm{Class}}_{h}}$.

A similar approach with the estimation of uncertainty in total AGC for stratification classes was used to estimate the uncertainty of the areas of each of the seven classes of forest disturbances and growth.

## Supplementary Material

Appendix 01 (PDF)

## Data Availability

Discrete airborne lidar transects data have been deposited in Zenodo (https://dx.doi.org/10.5281/zenodo.7636454; https://dx.doi.org/10.5281/zenodo.7689909) (87, 88).
