# Supplementary material for: A large net carbon loss attributed to anthropogenic and natural disturbances in the Amazon Arc of Deforestation
Source: Proc Natl Acad Sci U S A. 2024 Aug 5;121(33):e2310157121. doi: 10.1073/pnas.2310157121 (PMC11331119; doi:10.1073/pnas.2310157121)
Supplement: Supplementary file 1 — Appendix 01 (PDF) [file pnas.2310157121.sapp.pdf]

PNAS

Supporting Information for
A large net carbon loss attributed to anthropogenic and natural disturbances in the Amazon Arc of Deforestation

Ovidiu Csillik*, Michael Keller, Marcos Longo, Antonio Ferraz, Ekena Rangel Pinagé, Eric Görgens, Jean P. Ometto, Vinicius Silgueiro, David Brown, Paul Duffy, K.C. Cushman, Sassan Saatchi

* Corresponding author: Ovidiu Csillik
Email: ovidiu.csillik@gmail.com

This PDF file includes:

Figures S1 to S6
Tables S1 to S3

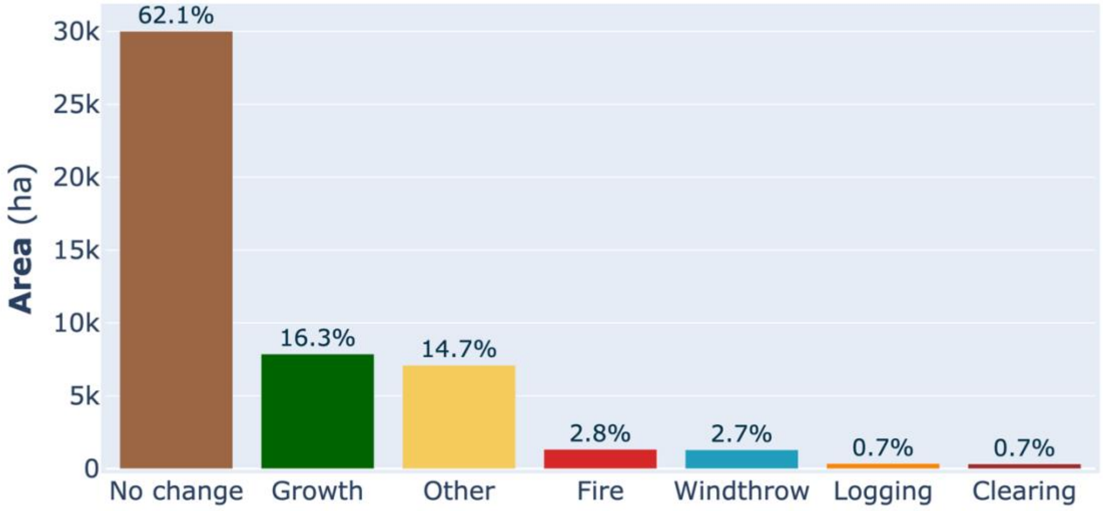

Fig. S1. Proportions of area for the seven classes within the 99 airborne lidar transects.

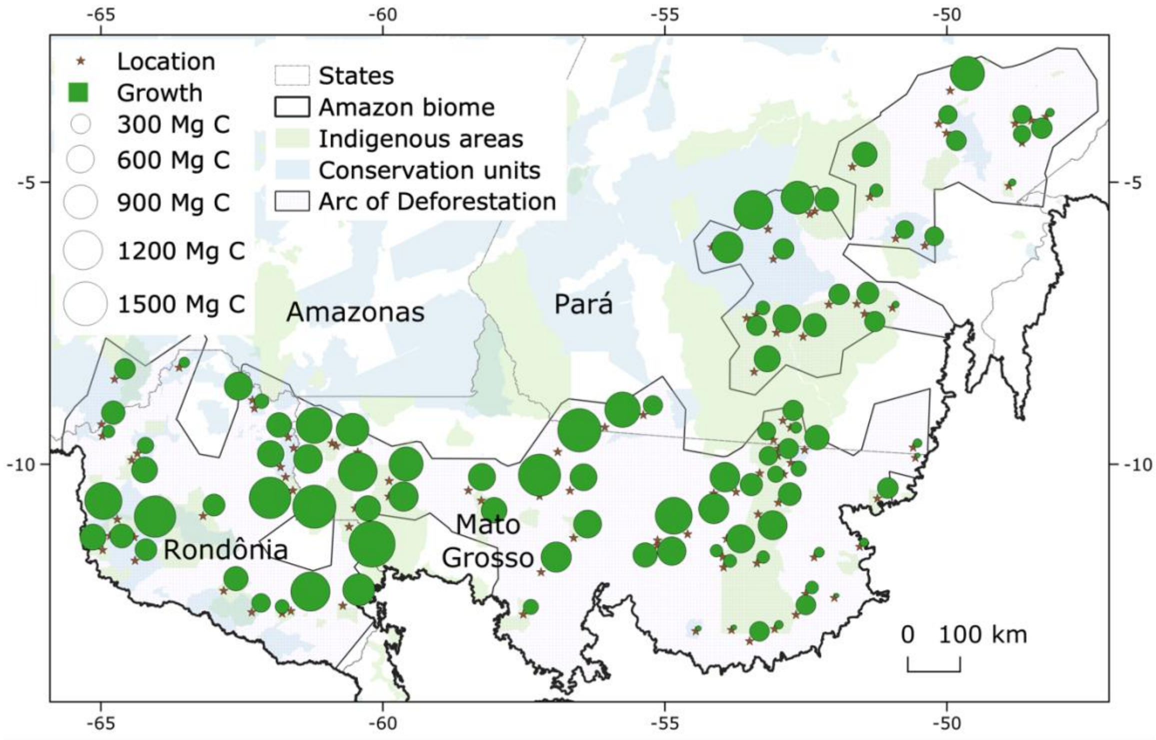

Fig. S2. Distribution and magnitude of carbon sequestration due to forest growth between the two lidar acquisitions. The size of the circles is related to the magnitude (range 16 - 1,676 Mg C).

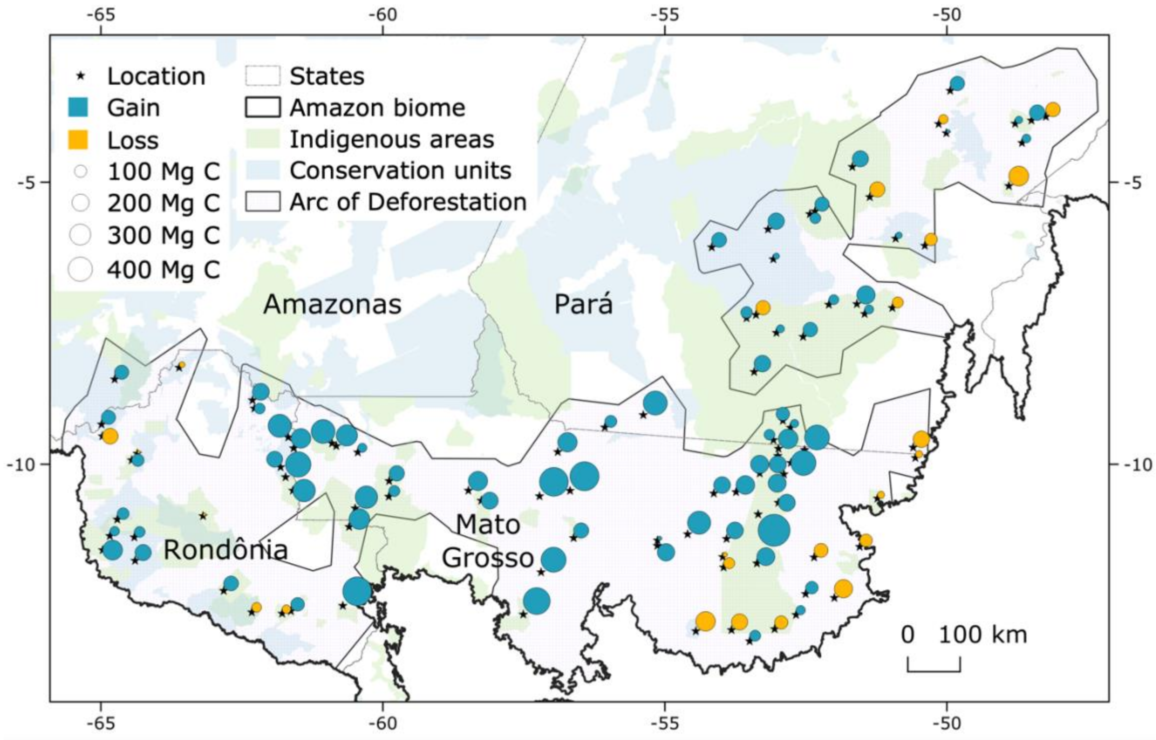

Fig. S3. Distribution of *no change* category, that comprises changes in canopy height smaller than 0.5 m between the two lidar campaigns. This class can include small forest growth and recovery, as well as low-intensity, small-scale disturbances. Most of the transects experienced small gains (blue circles, range 9 - 695 Mg C), compared to small losses (yellow circles, range 6 - 268 Mg C).

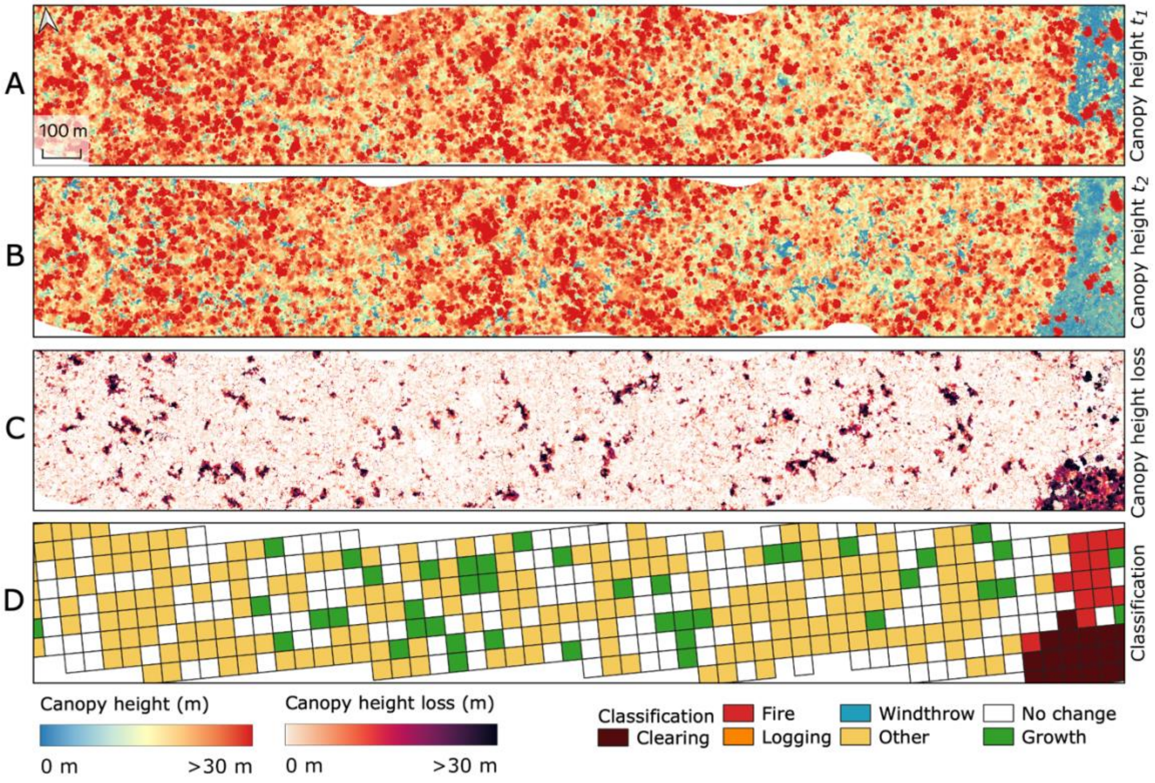

Fig. S4. Forest canopy height and height-loss patterns inside Karipuna Indigenous Territory in the state of Rondônia using canopy height models from two lidar campaigns. Illustrations show (A, B) canopy height of the two airborne lidar campaigns, (C) the canopy height loss between the two campaigns, and (D) the classification based on 50×50 m cells. We have conservatively classified selective logging that showed a combination of logging roads, logging decks, and removal of large trees. We detected a high rate of canopy disturbance that appeared to be anthropogenic inside the Karipuna Indigenous Territory. This disturbance lacked the pattern of conventional selective logging, specifically the absence of obvious roads for log transport. We conservatively classified this area within *other* disturbances.

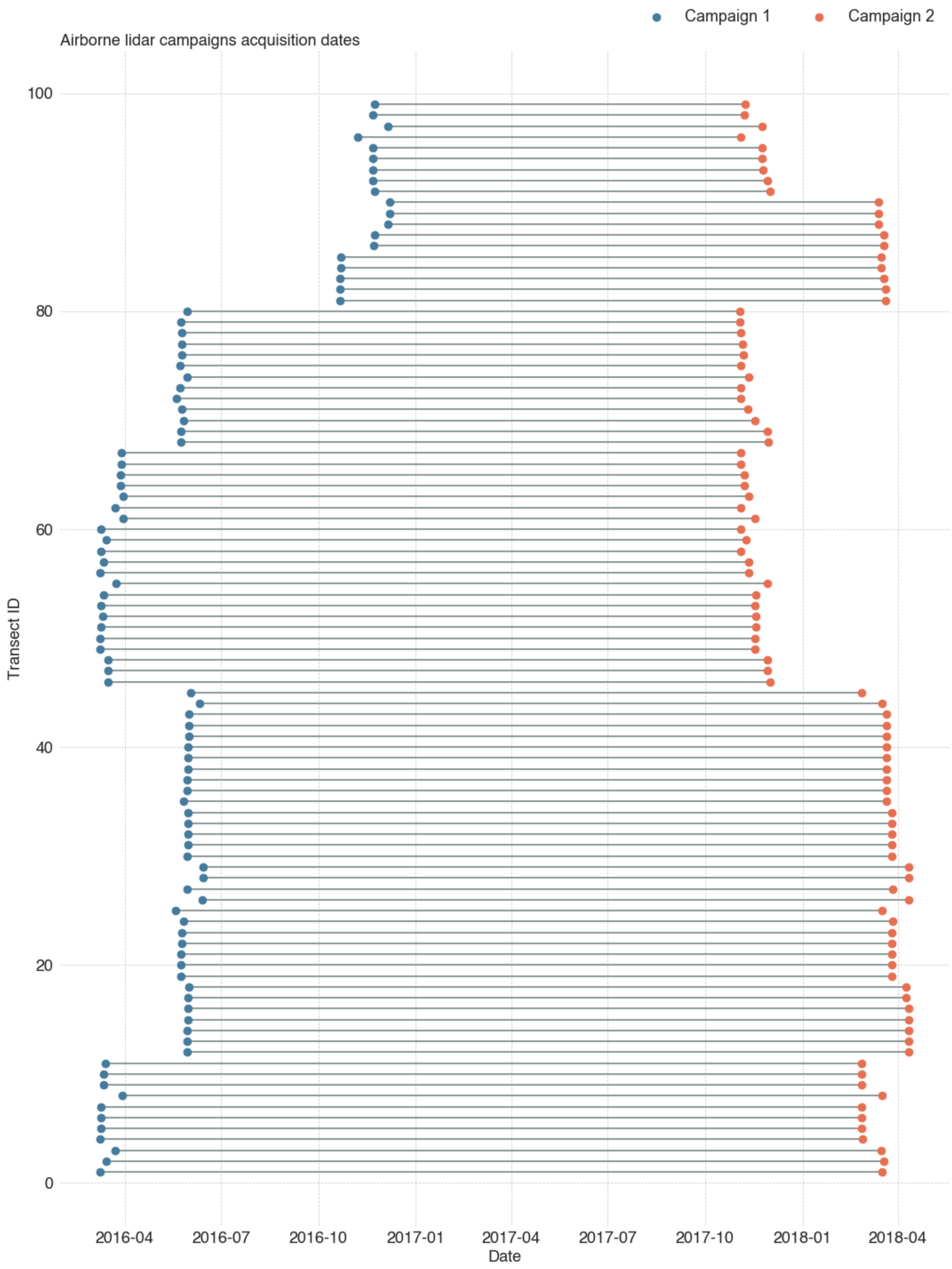

Fig. S5. Timeline of the two airborne lidar campaigns, ordered by the length of the time period between the two campaigns.

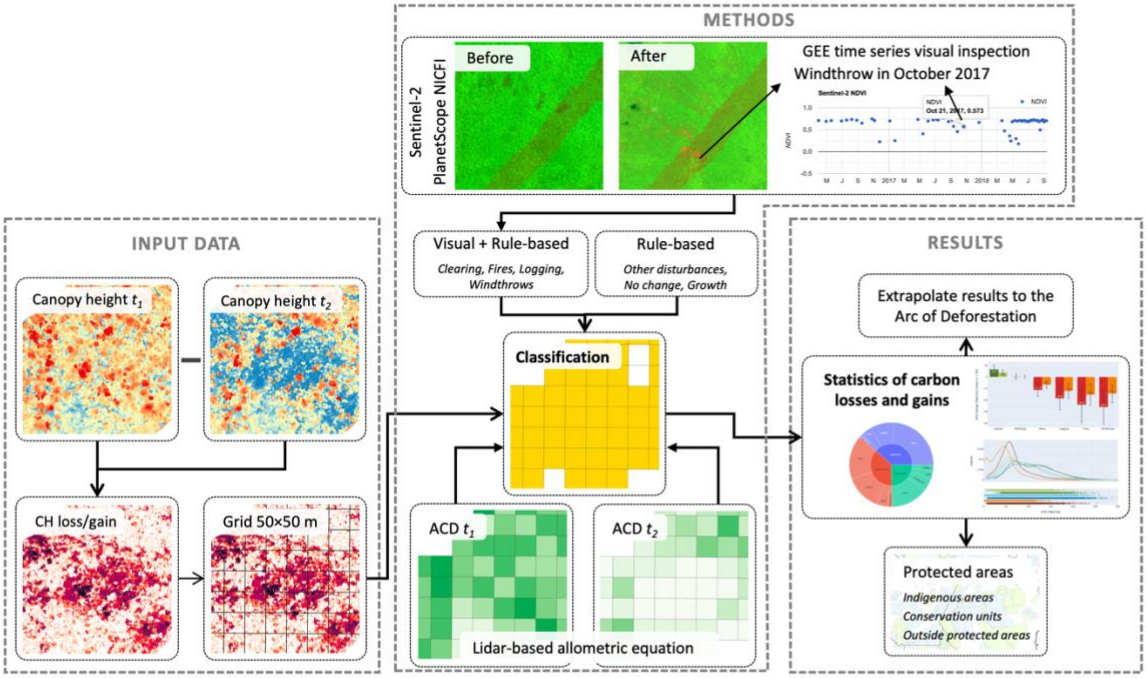

Fig. S6. Simplified methodological workflow using canopy height measurements from two different airborne lidar campaigns. We overlapped a grid of 50x50 m and estimated the aboveground carbon density (ACD) using a lidar-based allometric equation. Clearing, fires, logging, and windthrow events were identified by visual interpretation from Sentinel-2 and PlanetScope NICFI time series data in Google Earth Engine (GEE). We overlapped this identification with the 50×50 m grid and classified the cells into one of the four classes if they had a canopy height loss of more than 0.5 m (the vertical targeted accuracy of the lidar datasets). Other disturbances were classified as the cells with height losses of more than 0.5 m and not classified as clearing, fire, logging, and windthrow. Cells with height gains higher than 0.5 m were classified as growth, while no change class had height changes between -0.5 and 0.5 m. The results based on the 99 transects were extrapolated to the Brazilian Arc of Deforestation.

Table S1. A probabilistic confusion matrix between the seven classes of our classification. Values range from 0 to 1, with 0 depicting no confusion between classes and 1 denoting a perfect classification.

	**Growth**	**No change**	**Other**	**Windthrow**	**Fire**	**Logging**	**Clearing**
**Growth**	**1**	0	0	0	0	0	0
**No change**	0	**1**	0	0	0	0	0
**Other**	0	0	**0.80**	0.10	0.05	0.05	0
**Windthrow**	0	0	0.10	**0.80**	0.05	0.05	0
**Fire**	0	0	0.05	0.05	**0.80**	0.05	0.05
**Logging**	0	0	0.05	0.05	0.05	**0.85**	0
**Clearing**	0	0	0	0	0.05	0	**0.95**

Table S2. Confusion matrix for the selective logging class between our classification and SIMEX (Sistema de Monitoramento da Exploração Madeireira, https://imazon.org.br/?s=simex). We identified five transects with both our classification and SIMEX. One transect where we have classified logging SIMEX had a logging polygon nearby, but outside our lidar transect. In one lidar transect, SIMEX had logging and we had classified fire. In this case, we suspect that SIMEX used a late image of logging in July 2016 and identifying an event that occurred before our first lidar campaign. In one transect, SIMEX had few edge pixels classified as logging while we had fire. Producer's and user's accuracies for the logging classification were 83.0% and 74.4%, respectively.

Logging	**SIMEX Yes**	**SIMEX NO**
**Our classification YES**	1053	363
**Our classification NO**	216	191,508

Table S3. Confusion matrix for the forest clearing class between out classification and PRODES. We identified six transects with both our classification and PRODES. We had three transects detecting small clearing events. One transect had few edge clearing cells detected by PRODES while we have classified those cells as fire. Producer's and user's accuracies for the clearing classification were 95.2% and 54.3%, respectively. Our high-resolution approach detected more clearing events than the PRODES product that has a minimum mapping unit of 6.25 ha, hence the lower user's accuracy.

Clearing	**PRODES Yes**	**PRODES NO**
**Our classification YES**	707	596
**Our classification NO**	36	191,801
